# Cost analysis and environmental assessment of recycling paint sludge in asphalt pavements

**DOI:** 10.1007/s11356-020-10037-2

**Published:** 2020-07-13

**Authors:** Barbara Ruffino, Angela Farina, Davide Dalmazzo, Gianandrea Blengini, Mariachiara Zanetti, Ezio Santagata

**Affiliations:** Department of Environment, Land and Infrastructure Engineering, Politecnico di Torino, Corso Duca degli Abruzzi, 24, 10129 Torino, Italy

**Keywords:** Paint sludge, Automotive industry, LCA, Industrial costs, CO_2_ emissions

## Abstract

Paint sludge (PS) is a waste product coming from spray application of paints in automotive industry. For the first time, this work assessed the economic costs and environmental impacts connected to recycling PS in bituminous binders for asphalt pavement applications. Previous works have demonstrated that PS could be used as a replacement of up to 20% (w/w) of neat bitumen in the production of hot mixture asphalts (HMAs), without worsening the technical performances of pavements. The annual production of PS from Italian automotive plants (3000 t/year) could be accommodated in a paved area of 1.64 km^2^ that, when employed in local roads, with an average width of 5 m, corresponds to approximately 330 km. Costs for treating PS to be prepared for recycling resulted in 144 €/t raw PS. This cost was of the same order, or even less, of that required for PS incineration or disposal in a landfill for hazardous waste (250–300 €). The LCA analysis revealed that the production of HMAs by employing a binder that contains 20% (w/w) of PS, reduced the gross energy requirement (GER) and global warming potential (GWP) indexes by 15% and 39%, respectively, compared to an HMA produced with the traditional process.

## Introduction

Application of paints by spraying, extensively used in the automotive industry, is a significant source of solid waste. Currently, the generation of paint sludge (PS) in Italian plants is in the order of 3 kg/car on a wet basis (data from FCA 2019). PS composition depends on the substances contained in the used paints, but most part of PS is classified with EU waste code 080113*. This implies the presence of hazardous characteristics such as low flash point and substantial content of heavy metals, volatile organic compounds (VOCs), and other toxic substances that led to a high pollution potential (Gautam et al. 2010). Salihoglu and Salihoglu (2016) reported that PS generated by two Turkish automotive manufacturing plants represented a significant fraction (~ 35%) of the hazardous waste produced at the plant and their management cost accounted for almost 60% of the total environmental cost for hazardous waste management. Although past researches referred on landfilling of PS (Kim 2011), according to the EU Legislation, PS cannot be accepted by landfills, because of its high dissolved organic carbon (DOC) and total organic carbon (TOC) content. Presently, most part of PS is incinerated in special combustion plants or at licensed cement kilns as a refuse-derived fuel (RDF) for energy recovery (Salihoglu and Salihoglu 2016).

Solutions alternative to incineration or landfilling have been considered since the early 1990s, with the aim of reducing the impacts of PS on the environment and recovering valuable materials. For example, researchers from Ford company studied the technical feasibility of pyrolyzing PS to an activated carbon-like adsorbent that could be used to reduce the emissions of VOCs from spray booths (Kim et al. 2001). Recently, Li et al. (2018) assessed the possibility of using PS as a pore forming agent in the preparation of a sewage sludge-derived carbon for the adsorption of various contaminants. Other works considered the energy valorization of oils and gases extracted from PS by thermal processes such as pyrolysis, gasification, or liquefaction. Recently, Jayakishan et al. (2019) carried out a co-liquefaction of PS rich in hydrocarbons together with *Prosopis juliflora* biomass for bio-oil production. After the thermal treatment, the inorganic residues, made of metal oxides, could be recycled into the same source materials, which are paint fillers, from which they originated (Nakouzi et al. 1998; Khezri et al. 2012). Towards the end of the 1990s, ASTER Inc. proposed a solution to recycle PS into ingredients for automotive sealants (Gerace et al. 2002). In 2007, Indian researchers developed a treatment made of phases of consecutive rinsing with several solvents, drying, milling, and sieving for the conversion of PS into a reusable paint (Bhatia et al. 2007). In all the aforementioned case studies, waste products generated in car manufacturing processes were recycled into the same production chain.

More recently, some authors have proposed biological processes for recovering water-based PS (Tian et al. 2012a, b; Salihoglu et al. 2018). The high amounts of organic carbon and nitrogen and the low solvent content of water-based PS made such a waste product attractive to be composted or biodried. The recent study of Salihoglu et al. (2018) demonstrated that, although the concentration of some metals, like nickel (Ni) and tin (Sn), in the final composted product exceeded the legal compost limits, the calorific value made the composted waste suitable to be used as an additional fuel in cement kilns.

Another solution for recycling of PS is its addition in building materials, such as cement concrete, ceramics, or hot mix asphalts (HMAs). Feng et al. (2018) observed that both flexural and compressive strength of cement concrete increased when solid particles of PS were added at a level of below 10% of the cement weight. Meanwhile, the manufacturing of environmentally clean white ceramics was proposed to neutralize hazardous PS (up to 20 wt%) in kaolin clay composites (Mymrin et al. 2019). Dalmazzo et al. (2017) tested the reuse of dried and pulverized PS as a substitute of a part of the conventional binder for the production of HMAs for paving applications. In that study, carried out with the cooperation of an Italian automotive company, the characteristics of the binders modified with PS were carefully evaluated by using rheological tests (Dalmazzo et al. 2017). The authors concluded that the addition of amounts of PS up to 20% determined only minor changes in the physical properties of asphalt binders, in terms of performance grade (PG), elastic response, stress sensitivity, fatigue resistance, and fatigue ductility of neat bitumen. Furthermore, a subsequent study of the same authors (Zanetti et al. 2018) proved that the impacts on the environment of the phases of production of HMAs with a PS-modified binder and of the subsequent construction of the pavement were of limited extent. In fact, with an adequate tuning of the operating conditions of the recycling process (i.e., a drying process of PS carried out at a temperature value in the range 105–150 °C), the quality of the gaseous emissions generated during the phases of HMA production and lying did not relevantly differ from those obtained by producing and lying traditional, unmodified HMAs containing neat bitumen (Zanetti et al. 2018). Similarly, the presence of PS in the binder of an HMA did not negatively affect the quality of the leachate obtained by putting in contact asphalt concrete samples with water, according to the EN 12457/2 method (EN 12457-2 2002), with the aim of simulating the characteristics of runoff water.

However, in order to place the process of PS recycling in HMAs in a pre-industrial perspective, the laboratory investigation performed on the binder and HMAs obtained with the addition of PS must be supplemented with an economic analysis and an environmental assessment. The asphalt industry has recently recognized the LCA approach as the most effective tool for measuring and comparing the environmental performances and burdens of road pavements throughout their whole life cycle (Matthews et al. 2014; Gulotta et al. 2019). The LCA tool has been applied in a number of studies to evaluate and compare energy and environmental impacts of recycled (i.e., reclaimed asphalt pavement (Farina et al. 2017), crumb rubber (Puccini et al. 2019) and fibers (Landi et al. 2020) from end-of-life tires), and low-temperature materials (Praticò et al. 2020) employed for HMA production. In fact, one of the current challenges of the asphalt industry is to use recycled materials, such as PS, in partial replacement of bitumen, thus reducing carbon footprint and making the sector less dependent on petroleum-based products. As in the case of this study, the trend is to utilize by-products from industrial processes or waste products from the everyday life, avoiding their disposal in landfills, in accordance with the principles of sustainability and circular economy (Ingrassia et al. 2019).

In the light of above, this study is aimed to achieve two main results. On the one hand, it provides a detailed description of the economy costs involved in the process of recycling PS in asphalt pavements. The positive costs related with the operations that make PS recyclable and usable as a binder substitute are compared with the negative costs connected with avoided PS landfilling or incineration and binder saving, to finally demonstrate the economic feasibility of the recycling process. On the other hand, this study integrates the abovementioned state-of-the-art concerning the application of LCA to asphalt pavements, by comparing the environmental performance of a traditional HMA and that of an HMA that contains a PS-modified binder. Specifically, the environmental assessment is carried out in terms of global warming potential (GWP) and gross energy requirement (GRE), associated with the production phase of HMAs modified with PS, throughout a LCA. To the best of our knowledge, none of the processes presented in this introduction for the recovery/valorization of PS, the economy feasibility, and the energy/environmental compatibility was demonstrated by using the tools of the cost analysis and environmental assessment.

## Materials and methods

### Paint sludge characterization

This work was based on PS samples collected from six Italian automotive factories. Samples originated from painting operations carried out with primer, basecoat (water-based and solvent-based), and clearcoat paints. Details of sample characterization were provided in Dalmazzo et al. (2017). The main average characteristics of the PS samples are reported in Table 1, in which the total solid content and total volatile solid content are indicated by TS and TVS, respectively. The results of the PS characterization, in terms of moisture, metal content, and elemental composition, were in good agreement with those found in previous studies (Januri et al. 2015; Avci et al. 2017).

**Table 1:** Main characteristics of the PS samples considered in this work

Sample	TS (%)^a^	TVS (%)	C (%)	H (%)	N (%)	Fe (%)	Al (%)	Ti (%)
Primer (3)^b^	57 ± 22	61 ± 7	ND	ND	ND	0.9 ± 1.4	0.5 ± 0.2	8.3 ± 3.6
Basecoat (6)^b^	40 ± 9	73 ± 8	48 ± 6	6.4 ± 0.8	3.0 ± 1.4	0.5 ± 0.2	2.1 ± 0.9	7.4 ± 3.1
Clearcoat (3)^b^	35 ± 11	95 ± 1	60 ± 2	8.1 ± 0.1	7.5 ± 0.8	0.2 ± 0.2	1.0 ± 0.6	< 0.05

^a^The percentage of TS is referred to the raw sample; the percentage of all the other components is referred to the dried sample

^b^The number into parentheses refers to the total number of samples analyzed for each type of painting process operation

The utilization of PS as a binder modifier requires the samples to have good characteristics in terms of homogeneity and grindability to a fine powder (Dalmazzo et al. 2017). Because of their characteristics, only the PS samples coming from painting operations carried out with basecoat and clearcoat paints were found to be adequate for being treated, mixed with bitumen, and consequently were considered in this study.

### Process description

**Fig. 1:**
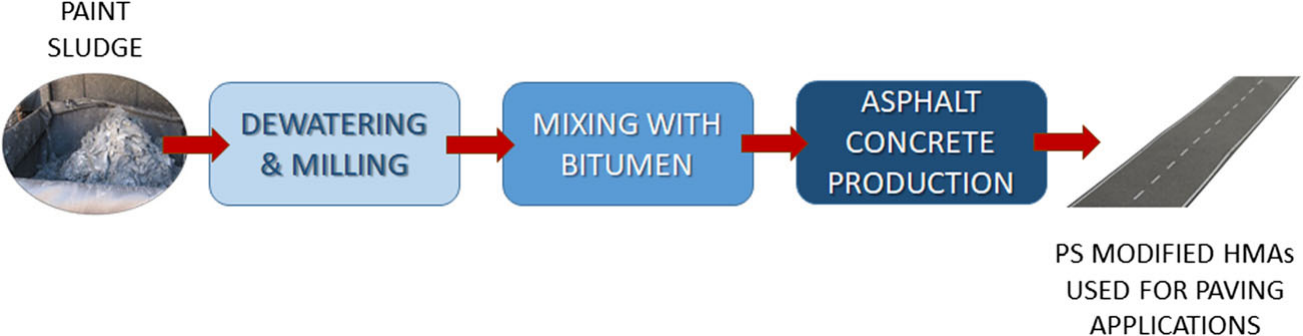
The PS recycling process. PS is firstly dewatered and milled, subsequently mixed with neat bitumen, and finally the modified bitumen is used to produce hot mix asphalts (HMAs) for paving applications

As shown in Fig. 1, unlike the traditional process of HMA production, the utilization of PS to modify neat bitumen requires two additional phases. These two phases include (1) PS preparation through the operations of drying and milling and (2) mixing of PS with the neat bitumen. In fact, PS must be dried and reduced to a fine powder before being used as an additive for asphalt binders.

#### Paint sludge preparation

**Fig. 2:**
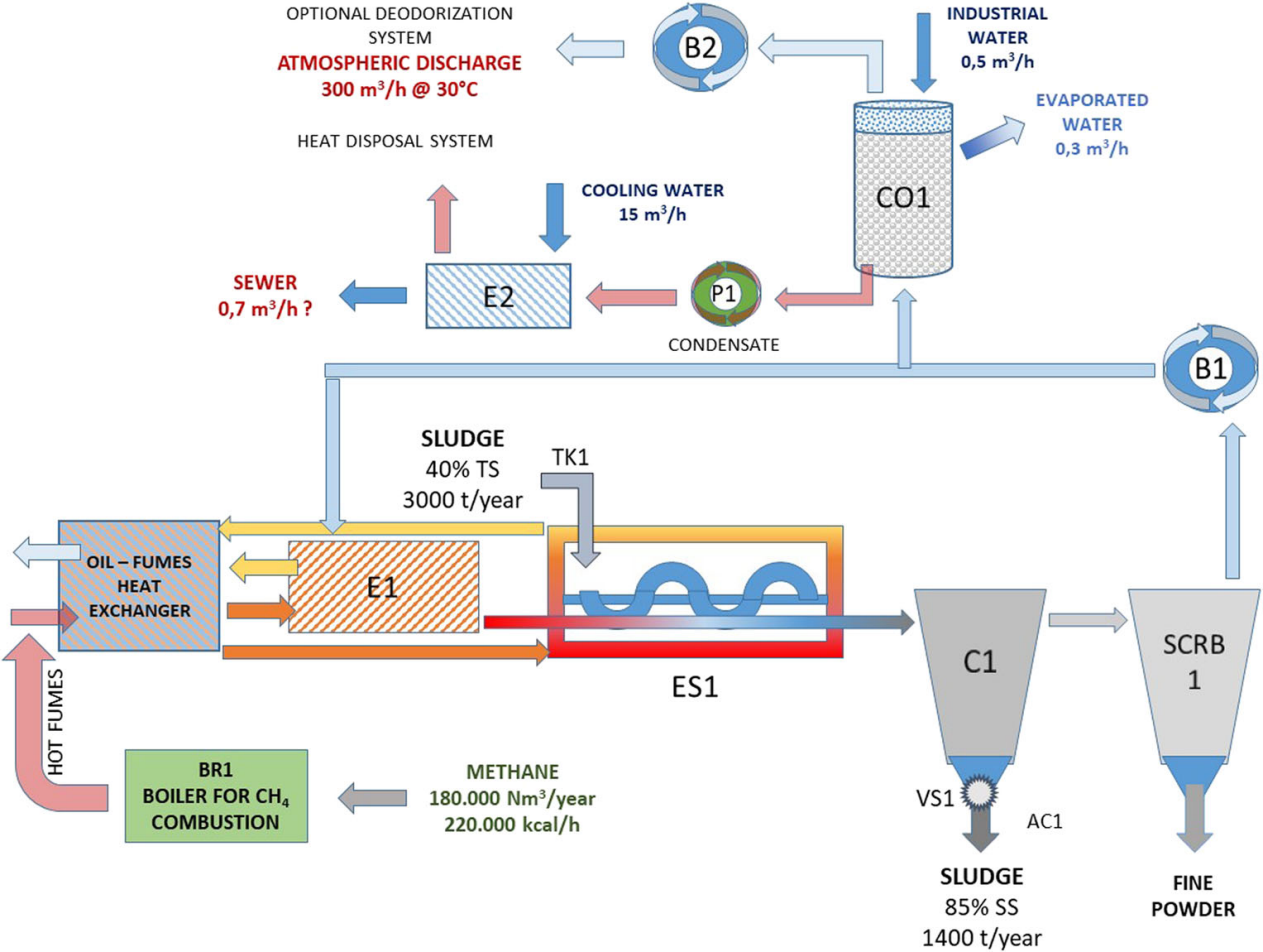
The turbo-drying apparatus for PS drying and milling

The phase of PS preparation takes place in a drying plant optimized for drying and crumbling the sludge. A scheme of the entire plant is provided in Fig. 2.

The core of the plant is the drying chamber labeled ES1. A co-current high-temperature vapor stream passes through the chamber and generates a homogeneous, high turbulent thin film of sludge. The drying chamber ES1 is equipped with a jacket and a centrifuge rotor; they have the purpose of holding the chamber walls at high temperature and distributing the sludge homogenously in the drier, respectively. The rotor, which rotates at a suitable angular velocity, maintains the sludge in the form of a thin film and moves it forward through the chamber. The drying process of the PS is aided by the contact between the wet sludge and the hot walls of the chamber. The average residence time of PS and vapor stream in the chamber is in the order of only 2 or 3 min.

Water is removed from PS in the form of vapor and the water content of sludge is lowered from 60 to approximately 10–15%. The dried product is subsequently recovered from the stream by a cyclone (C1). The residual fine powder is separated by a scrubber (SCRB1) and the low-temperature vapor is heated again in an oil–vapor heat exchanger (E1) to be reused as a sludge carrier and jacket filling. The extra amount of vapor is condensed in a condensation column (CO1) and, after a final cooling in a water–water heat exchanger (E2), discharged into the sewer. Dried sludge from C1 is ready to be mixed with neat bitumen.

#### Sludge mixing

The mixing process between the powdered PS, obtained as in the “Paint sludge preparation” section, and neat bitumen is carried out in a mixer with a capacity of 5500 l, a utilizable volume in the order of 4500–5000 l and working in a batch modality. According to the indications provided in Dalmazzo et al. (2017), the mixing phase must be carried out at 150 °C for 30 min. Moreover, since the obtained modified binder has a limited stability to storage, it must be produced immediately before utilization in HMAs (Dalmazzo et al. 2017).

### Evaluation of the recycling costs

**Fig. 3:**
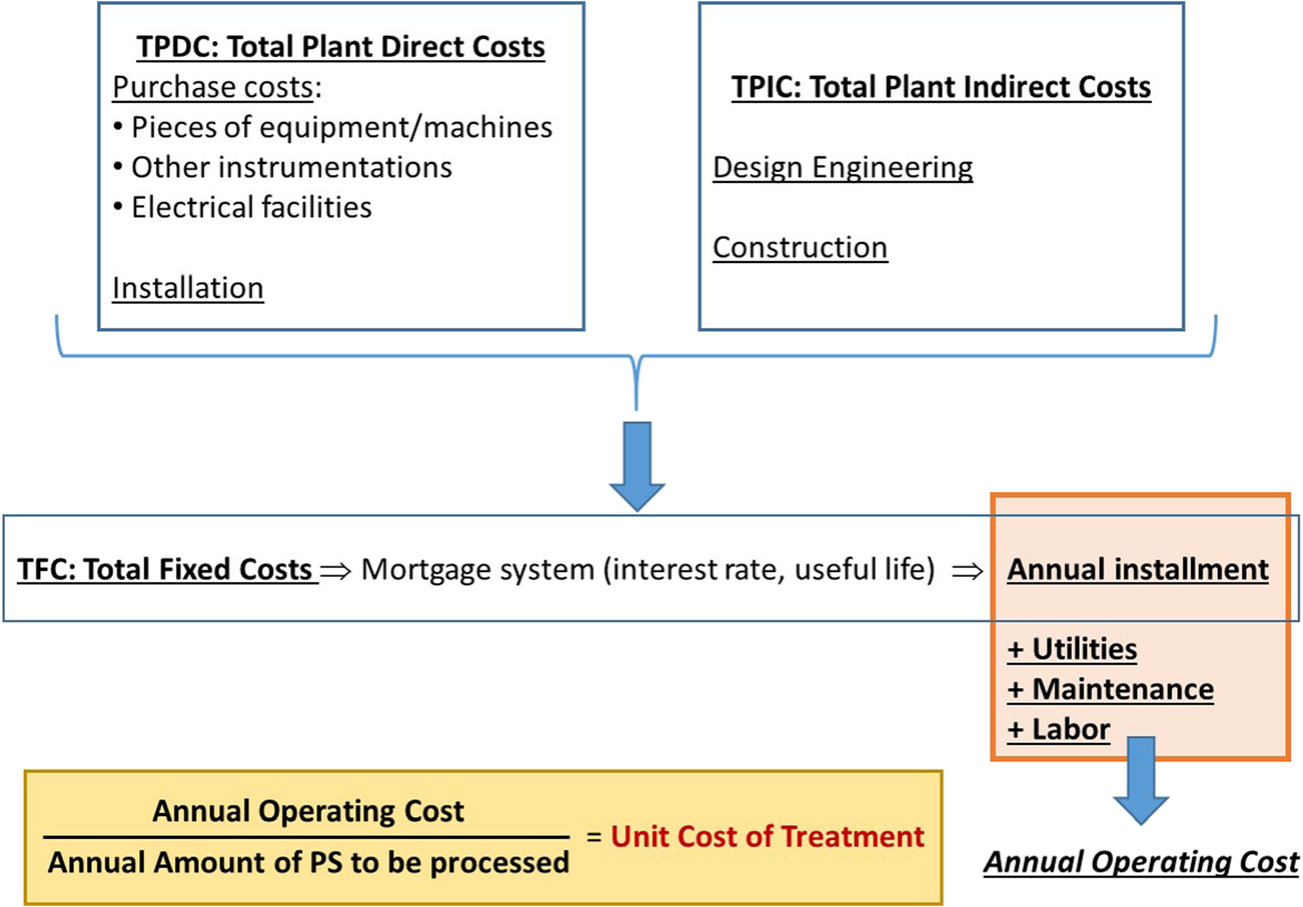
Main cost items for the process of PS recycling

The economic assessment of the recycling process of PS in HMAs was carried out by referring to a PS production rate of 3000 t/year, that is the average annual production of Italian automotive factories (data from FCA 2019). The method used for the evaluation of the costs of the recycling process was developed and optimized in previous studies concerning the recovery of secondary steel from grinding scraps (Ruffino and Zanetti 2008) and the valorization of useful fractions from exhaust portable batteries (Ruffino et al. 2011). Figure 3 summarizes the main cost items considered in this work.

As shown in Fig. 3, the final unit cost of treatment results from dividing the annual operating cost (AOC) by the amount of PS generated in 1 year that undergoes the treatment (3000 t). The AOC includes an annual installment coming from the mortgage of total fixed costs (TFCs) plus several items related to variable costs, such as utilities, maintenance, labor, and raw materials. TFCs are the sum of total plant direct cost (TPDCs) and total plant indirect costs (TPICs). As shown in Fig. 3, TPDCs include the costs for purchase and installation of the main pieces of equipment/machines, the minor instrumentations, and the electrical facilities. Conversely, TPICs include the costs for design and construction of the premises that will host the process at the industrial site. The TFCs are paid in a number of years through a constant annual installment which was calculated by multiplying the TFCs by the capital charge rate (CCR) (Ruffino and Zanetti 2017). The CCR is calculated as a function of the annual interest rate (*i*) and the operating lifetime (*n*) as described in Eq. (1)

1 $$ \mathrm{CCR}=\frac{i}{1-{\left(1+i\right)}^{-n}} $$

In this work, *i* and *n* were fixed to 6% and 10 years, respectively.

### LCA methodology

**Fig. 4:**
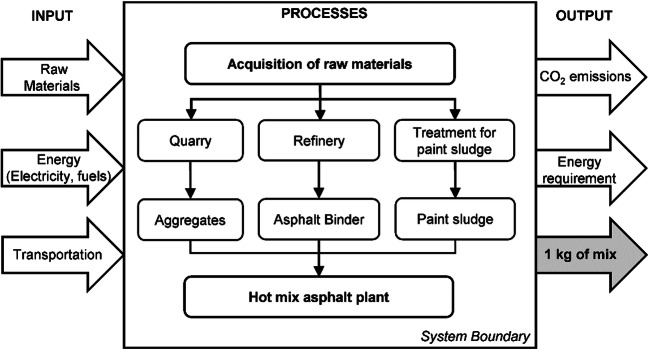
Boundaries of the system producing 1 kg of HMA

In this manuscript, the LCA approach was used to compare the environmental performances of an HMA containing a binder modified with PS, with a traditional HMA containing neat bitumen, through a so-called cradle-to-gate analysis. Since only the phase of materials’ production was considered in this study, the most appropriate functional unit, to which the results are referred, was 1 kg of HMA produced in a dedicated plant. As shown in Fig. 4, the system boundaries of the LCA included all processes involving raw materials sourcing, transportation to the HMA plant, and the asphalt concrete production.

LCA models have been implemented by using the SimaPro software, developed by Prè Consultant (SimaPro7 2006). Methods used to evaluate the environmental impacts were the gross energy requirement (GRE) and the global warming potential (GWP), based on Boustead Model 5.0 (Boustead and Hancock 1979) and IPCC 2006 method (IPCC 2006), respectively.

The life cycle inventory (LCI) is the phase of a LCA in which data are collected for modeling the unit processes related to all the activities involved in the analysis. In this specific case study, data referring to the production of materials, modified binder and, finally, HMAs, were collected from the available literature, through interviews with contractors and by using the Ecoinvent 2.2 database (Ecoinvent 2007). A list of the materials used to produce HMAs (aggregates and neat bitumen or aggregates and binder modified with PS) is provided in the following sub-sections as well as information on HMA preparation and details on the transportation processes of the materials involved in the analysis. Table 2 reports the quantities of the materials used to produce the two types of HMA.

**Table 2:** Quantities of materials included in 1 kg of hot mix asphalt

	Traditional HMA (kg)	Paint sludge HMA (kg)
Aggregates	0.95	0.95
Neat bitumen	0.05	0.04
Paint sludge	–	0.01

#### Aggregates

Crushed silica aggregates are an ingredient of both types of HMAs, produced with either neat or modified binder. Aggregates were supplied in four different size fractions (10/15, 3/8, and 0/5 mm and filler). Data related to the production of aggregates were taken from a LCA study that focused on the quarry’s activities in Piedmont region (NW Italy) (Blengini and Garbarino 2010). That study revealed that the production of 1 ton of aggregates, irrespective of the particle sizes of each fraction, required 5.3 kWh of electricity, 2.3 m^3^ of water, 0.005 l of lubricant oil, 0.02 kg of iron filters, 0.0014 kg of rubber for conveyors, and 0.31 l of gasoline (Blengini and Garbarino 2010).

#### Bitumen

Data from the Eurobitume report (2012) were used to create the process for asphalt binder production in the SimaPro software. The European study covers the bitumen production chain, cradle-to-gate. The stages involved in the production chain are crude oil extraction, transportation to Europe by pipeline and shipping, production, and storage. The production of 1 ton of neat bitumen required, as main inputs, 22.5 kg of natural gas, 50.5 kg of crude oil, 10.9 kg of coal, 0.03 kg of uranium, and 1239 l of water. According to the Eurobitume report (2012), 72 MJ/ton of modified asphalt was used as input data to mix the neat bitumen with the PS.

Based on ISO 14044 standard (), the feedstock energy is defined as the “heat of combustion of a raw material input that is not used as an energy source to a product system.” The inclusion of the feedstock energy in the analysis can sway the results of the study, because of the higher value of the energy assumed for the asphalt binder during its life cycle. Since the main objective of this study was the assessment of the environmental compatibility of the recycling process of a waste material like PS, the authors decided to exclude the feedstock component, leaving the possibility to provide a sensitivity analysis in a future work.

#### Paint sludge

The analysis included the energy required to make PS suitable to be mixed with neat bitumen. That amount of energy was equal to 120 kWh of electricity and 60 m^3^ of methane for drying and milling 1 ton of PS (data provided from the company that produces and commercializes the turbo-drying apparatus for PS drying and milling).

The recovery process of PS allowed avoiding incineration. For this reason, the avoided transport to the incinerator plant and the process of incineration, as well, were included in the analysis by using the Ecoinvent process “Disposal, hazardous waste, 25% water, to hazardous waste incineration” (Ecoinvent 2007), which considers air and water emissions from the incineration of hazardous waste products.

#### Hot mix asphalt

Data related to the energy used to produce HMAs were collected from a plant located in the Piedmont region. It was assumed that for 1 ton of a traditional HMA, produced at 160 °C, the average annual energy consumption counts 9.8 m^3^ of methane to dry aggregates, 1 m^3^ of methane to heat the bitumen tank, and 4.25 kWh of electricity to mix ingredients. The same data were considered in the scenario that involves the HMA containing PS.

#### Transportation

To model the transportation phase, the Ecoinvent process “Transport, lorry 16-32t, EURO4” (Ecoinvent 2007) was used. The distances taken into account in this study, and listed in Table 3, have been evaluated by assuming the production of HMA in an industrial site located in Piedmont region .

**Table 3:** Distances covered by road transportation

Material/Process	Origin/destination	Distance (km)
Aggregates	From quarry to HMA plant	20
Bitumen	From refinery to HMA plant	350
Paint sludge	From automotive plant to HMA plant	7
Incinerator	From automotive plant to incinerator	10

## Results and discussion

### Economic assessment

The annual operating cost items (i.e., installment, utilities, maintenance, and labor) of the two phases of sludge preparation and mixing with neat bitumen were calculated in order to obtain an overall unit treatment cost.

#### Sludge preparation

The moisture content of PS is a crucial problem that affects the cost and convenience of sludge recovery (Yenikaya et al. 2018; Li et al. 2019). Sludge must be dried to decrease its volume and make it suitable to be recycled as an additive of neat bitumen. As described in the “Paint sludge preparation” section, dewatering and milling at a full scale are jointly carried out in a turbo-drying apparatus capable of reducing the original PS water content, equal to approximately 60% (w/w, on a wet basis), to values of less than 20%. Based on the data provided by the company that produces and commercialize the drying and pulverizing unit, the cost of a drying centrifuge with a capacity of 3000 t/year was in the order of 1100 k€. The same company estimated the other direct and indirect costs, necessary to prepare the site to receive the machine, in the order of 10% of the purchase cost (approximately 110 k€). Consequently, the TFCs resulted in 1210 k€ and the annual installment of 164.4 k€.

The thermal power necessary for water evaporation was quantified in 220 Mcal/h. The thermal power was obtained from the combustion of methane and transferred to the drying chamber ES1 in the form of high-temperature vapor. A volume of 31 Nm^3^/h (or 180,000 Nm^3^/year) of methane was required to feed the boiler BR1. Other than the heat, the drying centrifuge required 360 MWhe/year of electric power. Methane and electric power supplied for industrial uses were included in the utilities costs, with a unit cost of 0.3 €/Sm^3^ and 0.1 €/kWhe, respectively. Considering the amounts of methane and electric power required for the process, the total cost for utilities resulted in 90 k€/year.

According to the data provided by the company, the maintenance of the drying centrifuge could be accounted in 2% of its purchase cost, that is 22 k€/h. The process required supervision and maintenance for an average time of 2 h/day that resulted in 0.25 workers per day with a consequent annual cost of 9 k€. As detailed in Table 4, the sum of the purchase costs of the main pieces of equipment that constitute the drying plant and of the operating costs for maintenance, labor, electricity, and methane supply, returned an annual operating cost of 285 k€ and a consequent unit cost of 95 €/t for the phase of sludge drying and pulverization. The performed cost analysis did not take into account the costs for diathermic oil substitution (in the oil–fumes heat exchanger) and for industrial water because it accounted for less than 1% of the installment cost.

**Table 4:** Cost items for the drying process

Cost item	Number of needed units	Unit cost (k€/unit)	Total cost (k€)
Installment	–	–	164.4
Maintenance	0.02	1100	22.0
Labor	0.25 operators	36	9.0
Electrical energy	360,000 kWhe	0.1	36.0
Methane	180,000 Sm^3^	0.3	54.0
Annual operating cost			285.4

#### Sludge mixing

The residual amount of PS after the drying and milling phase was in the order of 1400 t/year. The mixing process of pulverized PS with neat bitumen required a mixer with a working volume in the order of 5 m^3^ and a capacity of 235 kg/h (operating time, 6000 h/year). One of the most relevant feature of the mixer is the presence of a jacket containing heated oil with the purpose of guaranteeing the temperature of 150 °C during the mixing phase.

For the evaluation of the costs of the mixing phase, it was assumed that the PS content in the modified binder could range from 10 to 20% (w/w). Values in that range proved to be the best based on the results of the tests carried out at a lab scale with PS amounts from 0 to 20% (Dalmazzo et al. 2017). The data concerning the purchase cost of the mixer and the costs for energy, maintenance, and labor were provided by the company, that produces asphalt concretes for paving applications, mentioned in the “Hot mix asphalt” section.

The purchase cost of the mixer was in the order of 54 k€. As in the case of the drier, the other direct and indirect costs necessary to prepare the site to receive the machine were estimated in the order of 10% of the purchase cost (approximately 5.4 k€). Consequently, the TFCs resulted in 59.4 k€ and the annual installment of 8.1 k€.

The process required the continuous supervision of a skilled worker, with a consequent annual cost of 36 k€. Maintenance costs were quantified by taking into account that, according to the recommendations of the company, the mixer must be cleaned once a week. The cost of each cleaning operation was of 1.5 k€. The same company stated that the production of 1 ton of modified binder costs 14 k€ for electric energy and 14 k€ for the methane necessary to heat the mixture. The sum of costs, detailed in Table 5, to produce a modified binder from neat bitumen and pulverized PS was of 147.1 k€. The unit cost of this phase, referred to the original annual amount of PS (3000 t), was in the order of 49 €/t.

**Table 5:** Cost items for the mixing process

Cost item	Number of needed units	Unit cost (k€/unit)	Total cost (k€)
Installment	–	–	8.07
Maintenance	50	1.5	75
Labor	1 operator	36	36
Electrical energy	14,000 kWhe	1	14.0
Methane	14,000 Sm^3^	1	14.0
Annual operating cost			147.1

#### Comprehensive cost evaluation and full-scale implementation

Based on the results presented in the “Sludge preparation” and “Sludge mixing” sections, the unit cost of treatment for PS recycling, which includes the operations of sludge drying and pulverization and the subsequent mixing with neat bitumen, resulted in 144 €/t. The work of Dalmazzo et al. (2017) demonstrated that a dried and milled PS could substitute up to 20% of neat bitumen in a binder used for HMA production, without worsening the performances of the pavement.

The economic benefits related to the recycling process of PS and the consequent availability of the treated PS for bitumen modification could be assessed by considering the following:

• The avoided cost of sludge landfilling or incineration, which in the European countries presently accounts for 250–300 €/t

• The avoided cost of bitumen supply, due to the substitution with PS. Such a cost coincides with the average cost of bitumen that in this study was assumed equal to 400 €/t, according to the current trend of the Italian market

**Fig. 5:**
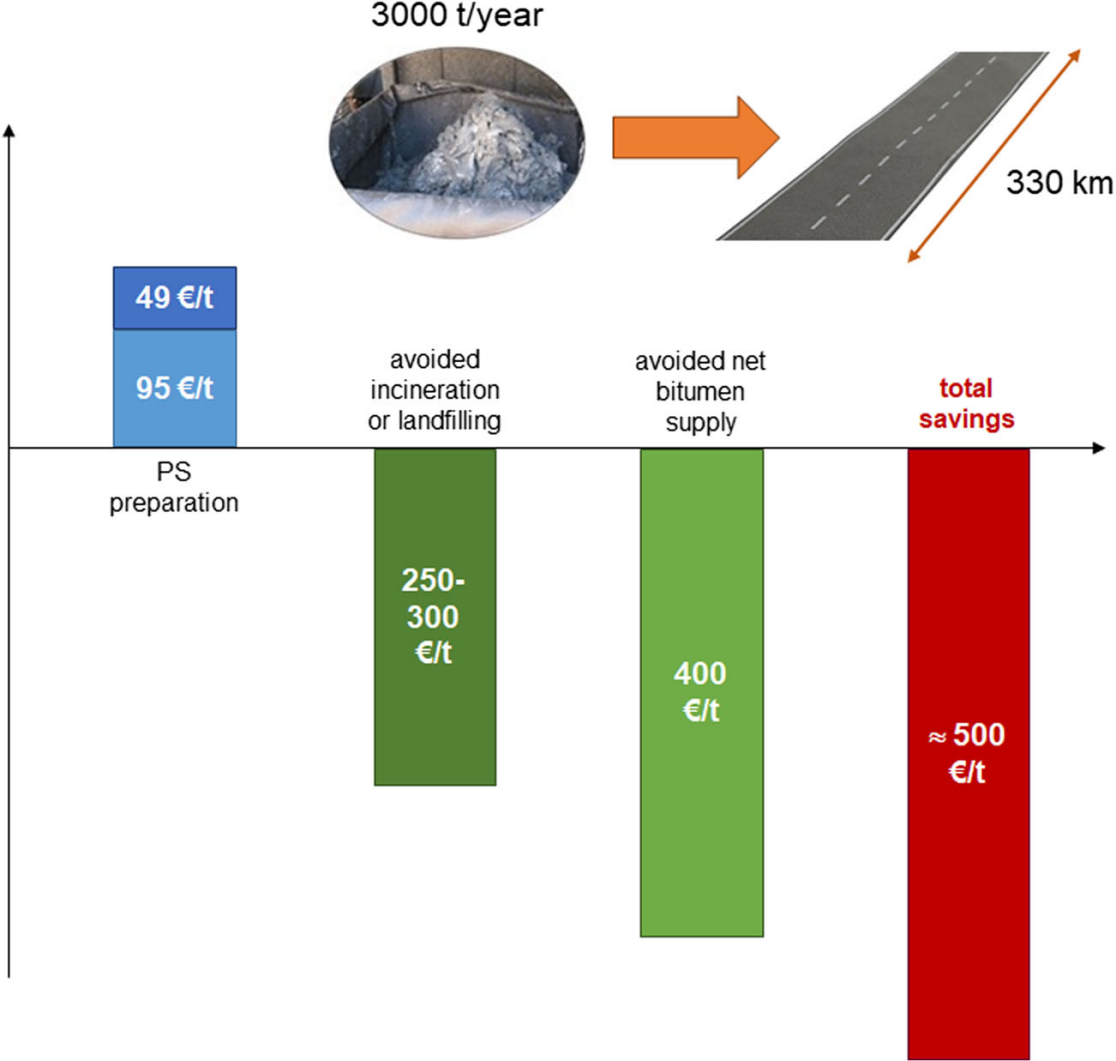
Resume of the cost analysis results and implementation of the PS recycling process at a full scale

The above-described analysis returned a total saving of approximately 500 € for each ton of raw PS, as detailed in Fig. 5. Such a saving will be distributed among the various actors of the future scenario that will result from an industrial implementation of the process for PS recycling. The involved actors are the automotive industry, which provides the waste product, the company which makes the sludge ready for binder preparation and, finally, the company specialized in the production of HMA for paving applications which uses the PS-modified binder. Effective savings may also be affected by other factors not considered in this analysis which may depend upon local constraints and commercial strategies.

The significance of this study can be assessed by referring to the extent of paving works which can potentially receive the annual production of PS of the Italian automotive plants (3000 t/year). Such an estimate is dependent upon a number of factors such as the PS dosage in the bitumen, the binder content and composition of HMAs, and the thickness of the wearing course layer and road width. Based on the results from Dalmazzo et al. (2017), the dosage of PS and the binder content were fixed to 20% and 5.3% w/w, respectively. By using the data listed in Table 6, a volume of HMA of approximately 50,000 m^3^ could be obtained from an original PS amount of 3000 t.

**Table 6:** Data used for the estimation of the surface and the road length paved with a HMA containing PS-modified binder

Average PS dosage in the binder (% w/w)	20
Binder content in the HMA (% w/w)	5.3
HMA density (kg/m^3^)	2300
Thickness of the wearing course (m)	0.03
Width of the road (m)	5

In a future scenario, HMAs containing PS-modified binder will be mainly used for maintenance purposes of different road categories, with an average width of 5 m, in the local/provincial network. It was calculated that the treated PS could be included in paving works for a total paved area of 1.64 km^2^ and a total road length of approximately 330 km.

### LCA analysis

**Fig. 6:**
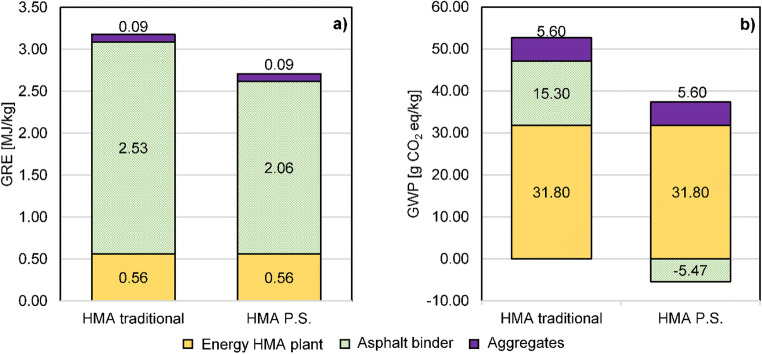
Contribution of hot mix asphalt components to **a** gross energy requirement (GRE) and **b** global warming potential (GWP)

The results of the LCA analysis are shown in Fig. 6. The GER index refers to the overall energy spent for the process of HMA production with either neat bitumen or PS-modified binder. The GWP quantifies the emissions of carbon dioxide (CO_2_) into the atmosphere and, consequently, the impact of the abovementioned processes on climate change.

The LCA analysis revealed that the production of an HMA by employing a bitumen with the addition of 20% (w/w) PS, reduced the GER and the GWP by 15% and 39%, respectively, compared to the HMA produced with the traditional process. Specifically, the GER index decreased from 3.18 to 2.71 MJ/kg and the GWP from 52.7 to 31.9 g CO_2_eq/kg of produced HMA. The use of PS as an asphalt binder modifier allowed to decrease the carbon footprint by approximately 21 g CO_2_eq/kg of HMA produced. Figure 6 details the contributions of each HMA component (aggregates, bitumen, and energy at the HMA plant) in terms of energy required during the manufacturing phase and regarding carbon dioxide emissions, respectively.

In agreement with the results of Santero et al. (2011), this study proved that the phase of binder production in both scenarios was the most energy intensive process. That phase accounted for 80% and 76% of the overall energy spent for HMA production, for modified binder and neat bitumen respectively. Conversely, the highest contributions to CO_2_ emissions was due to the phase of binder mixing with aggregates at the plant.

Since the quantity of the aggregates used to produce HMA with the two binders and the energy consumption for the mixing phase at the HMA plant were the same in the two considered scenarios, the only difference was found in the bitumen contribution. Results depicted in Fig. 6 show that the environmental benefit coming from the recovery of PS as a recycled component in HMAs overcame the energy requirement in terms of MJ consumption. The negative value associated with the impact of the binder modified with PS (− 5.47 g CO_2_eq/kg HMA) indicated that the environmental burdens related to the treatment of PS through the operations of drying and milling were lower than the benefits coming from the avoided emissions of the incinerator and the lower amount (− 20%) of neat bitumen used in HMAs.

## Conclusions

This work analyzed for the first time the economic costs and environmental impacts associated with the recycling process of PS as a modifier agent of the binder used in HMAs for paving applications. Previous studies demonstrated that the substitution of neat bitumen with up to 20% w/w of PS in the HMA binder did not worse the technical performances of the pavement and did not generate relevant impacts on the environment in terms of gaseous emissions and quality of runoff waters.

The annual production of PS from Italian automotive plants (3000 t/year) could be accommodated in 1.64 km^2^ of asphalt pavements that, when employed in local roads, with an average width of 5 m, corresponds to approximately 330 km. However, the employment of PS in HMAs for paving applications requires a preliminary phase of drying and milling that must be carried out in a dedicated turbo-drying apparatus. The cost of the treatment made to dry and pulverize PS was estimated to approximately 140 €/t raw PS. This cost was of the same order, or even less, of that required for PS incineration or disposal in a landfill for hazardous waste (250–300 €/t). Moreover, the actual saving of virgin bitumen (400 €/t) made PS an attractive low-cost substitute.

The LCA analysis revealed that the production of HMA by employing a binder that contains 20% (w/w) of PS required a GER of only 2.71 MJ/kg HMA, compared to 3.18 MJ/kg HMA necessary to the traditional process, with a saving of 15%. Emissions of CO_2_ were reduced by 39%, passing from 52.7 g CO_2_eq/kg HMA of the traditional process to 31.9 g CO_2_eq/kg HMA of the process that employs the binder with 20% PS.
